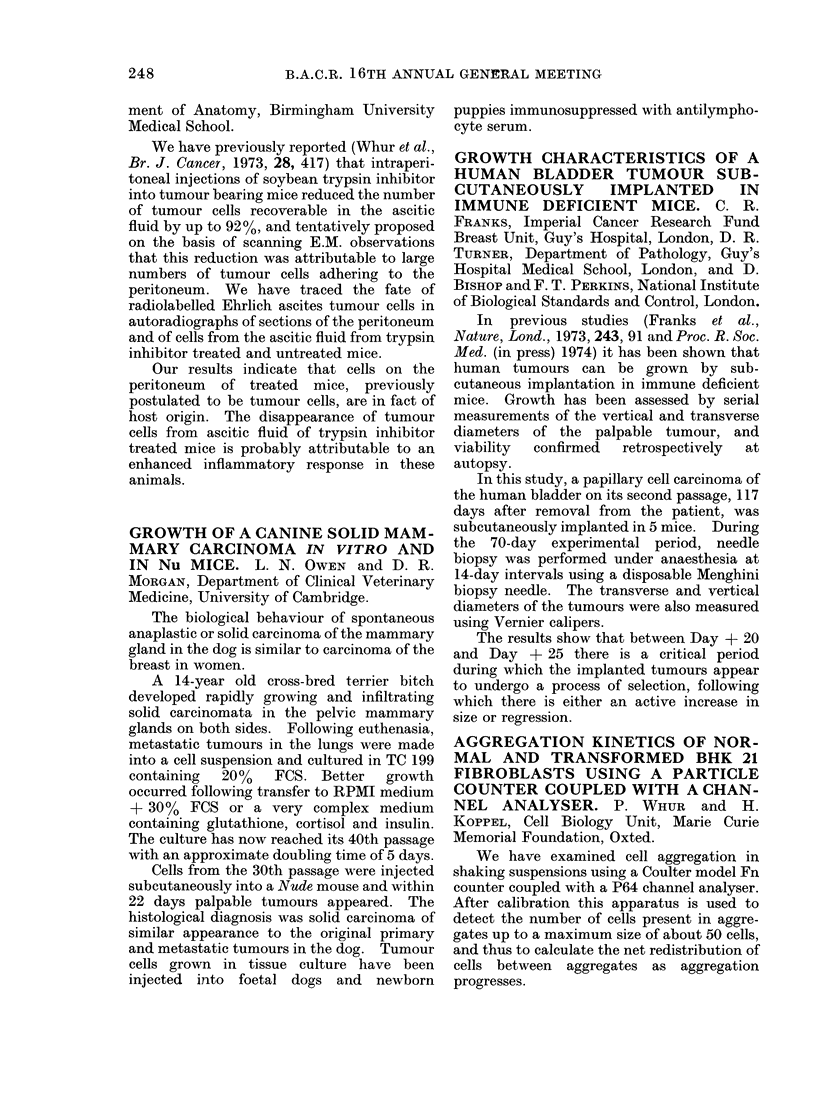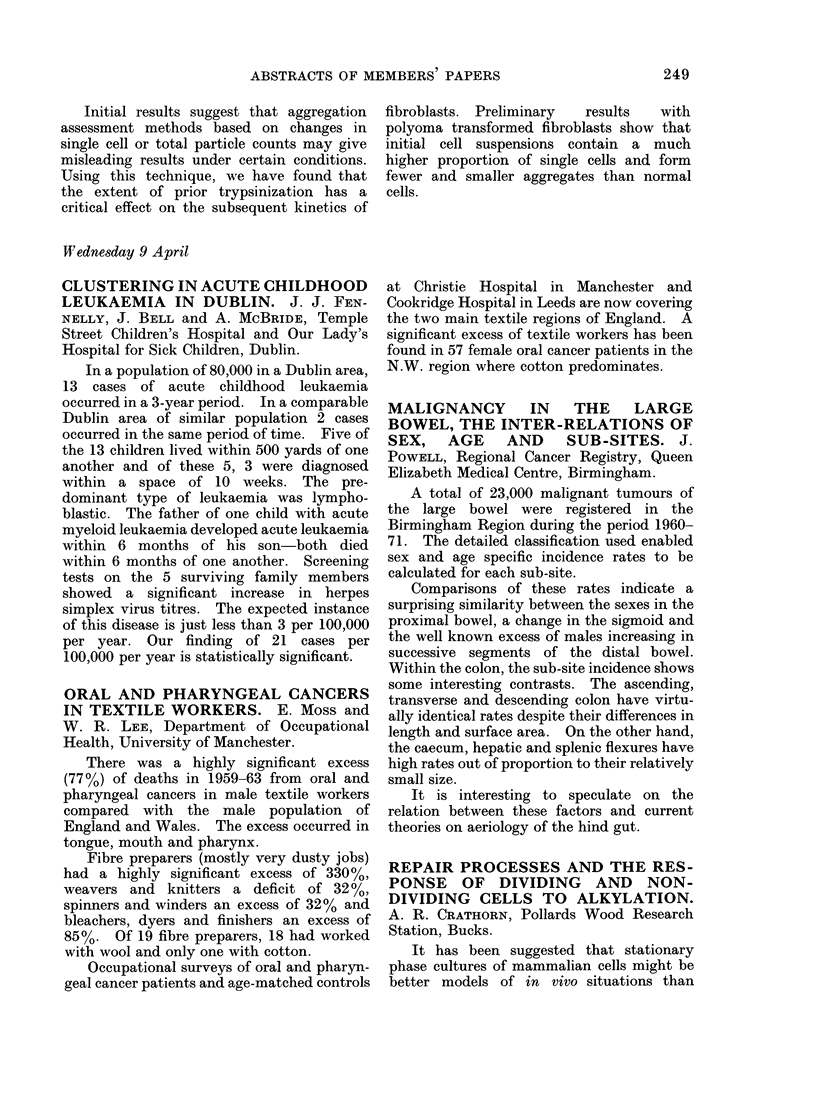# Proceedings: Aggregation kinetics of normal and transformed BHK 21 fibroblasts using a particle counter coupled with a channel analyser.

**DOI:** 10.1038/bjc.1975.183

**Published:** 1975-08

**Authors:** P. Whur, H. Koppel


					
AGGREGATION KINETICS OF NOR-
MAL AND TRANSFORMED BHK 21
FIBROBLASTS USING A PARTICLE
COUNTER COUPLED WITH A CHAN-
NEL   ANALYSER. P. WHUR         and  H.
KOPPEL, Cell Biology Unit, Marie Curie
Memorial Foundation, Oxted.

We have examined cell aggregation in
shaking suspensions using a Coulter model Fn
counter coupled with a P64 channel analyser.
After calibration this apparatus is used to
detect the number of cells present in aggre-
gates up to a maximum size of about 50 cells,
and thus to calculate the net redistribution of
cells between aggregates as aggregation
progresses.

ABSTRACTS OF MEMBERS PAPERS                    249

Initial results suggest that aggregation
assessment methods based on changes in
single cell or total particle counts may give
misleading results under certain conditions.
Using this technique, we have found that
the extent of prior trypsinization has a
critical effect on the subsequent kinetics of

W1'ednesday 9 April